# Sequence and phylogenetic analysis of the mitochondrial genome for the groundhopper *Mazarredia convexa* (Orthoptera: Tetrigidae)

**DOI:** 10.1080/23802359.2020.1772137

**Published:** 2020-06-02

**Authors:** Xiao-Dong Li, Wei Zhang, Lei Xin, Rong Ye, Wei-an Deng, Ran Li

**Affiliations:** a School of Chemistry and Bioengineering, Hechi University, Yizhou, P.R. China;; b The Key Laboratory of Jiangsu Biodiversity and Biotechnology, College of Life Sciences, Nanjing Normal University, Nanjing, P.R. China

**Keywords:** Mitogenome, Orthoptera, Tetrigidae, *Mazarredia convexa*, phylogenetic analysis

## Abstract

Using next generation sequencing (NGS), we determined the mitochondrial genome of *Mazarredia convexa*. The assembled mitochondrial genome of *M. convexa* was found to be 15,089 bp, consisting of 37 genes (13 protein-coding genes, 22 tRNA genes, and two rRNA genes). The region that we failed to sequence was between *rrnS* and *trnI*, and generally contained a putative AT-rich region. Its gene composition and order were similar to all reported tetrigid species. The overall nucleotide composition was 43.4% of A, 30.6% of T, 9.4% of G, and 16.6% of C. The data can help to better understand the phylogenetic status of *M. convexa* in Tetrigidae.

The genus *Mazarredia* was erected by Bolivar in 1887. Here considered a member of the subfamily Metrodorinae (Orthoptera: Tetrigidae). This genus currently includes 48 known species, which are mainly distributed in Cameroon, Vietnam, Malesia, Philippines, India, Indonesia, and China (Deng 2016). However, there is few information on its systematic position within Tetrigidae. To date, no mitochondrial and ribosomal sequences have been reported for Metrodorinae (NCBI, last visited on February 2020). To further advance evolutionary studies for Metrodorinae, we sequenced and analyzed the mitochondrial genome of *Mazarredia convexa* Deng, Zheng & Wei, 2007, which is the first mitogenome sequence in Metrodorinae.

In the current study, the samples of *M. convexa* were collected from Mei county in Guangdong province, China. And the voucher specimen was preserved in the Museum of Insects of Hechi University (the voucher No. O202). Total genomic DNA was obtained from the legs of an adult specimen using a Wizard^®^ Genomic DNA Purification Kit (Promega, Madison, USA) according to the manufacturer’s instructions. The genomic DNA was then sequenced using the Hiseq2500 platform (Illumina Inc., San Diego, CA). The extracted DNA was preserved at –20 °C in the Museum of Insects of Hechi University. The mitogenome was assembled with Geneious 9.0.4 (Kearse et al. 2012), annotated with MITOS Web Server (Bernt et al. 2013), and deposited in GenBank with the accession number MN938924.

Our mitochondrial assembly of *M. convexa* has a length of 15,089 bp, containing 13 protein-coding genes (PCGs), 22 tRNAs, and two rRNA unit genes (*rrnL* and *rrnS*). The region that we failed to sequence was between *rrnS* and *trnI*, and generally contained a putative AT-rich region. The overall nucleotide composition was 43.4% of A, 30.6% of T, 9.4% of G, and 16.6% of C. Nine PCGs and 14 tRNA genes were transcribed from the majority strand, while the remaining four PCGs (*ND1*, *ND4*, *ND4L*, and *ND5*), eight tRNAs and two rRNAs were located on the minority strand. In addition, the gene composition and order were similar to all reported tetrigid species. For the 13 PCGs, 10 PCGs started with typical ATN codon (two with ATC, two with ATA, six with ATG), whereas the *ND3*, *ND4L*, and *ND6* genes appeared to start with GTA, TTA, and TTG, respectively. Twelve PCGs ended with complete stop codons (two with TAG, 10 with TAA), and *ND5* ended with the incomplete stop codons T (TA–), which were presumably completed as TAA by post-transcriptional polyadenylation (Anderson et al. 1981).

The phylogenetic relationship was constructed with two methods: Bayesian Inference (BI) using MrBayes 3.1.2 (Huelsenbeck and Ronquist 2001) and Maximum-Likelihood (ML) using RAxML 8.2.0 (Stamatakis 2014), based on 13 PCGs from mitogenomes of eight tetrigid species and one outgroup, respectively. Two phylogenetic trees using different methods yielded the same topology, and nodal supporting values were always higher for BI tree than for ML tree (Figure 1). The phylogenetic analyses showed that the monophyly of the subfamily Tetriginae was strongly supported. The clade of *M. convexa* is a sister clade to the clade of *Thoradonta obtusilobata* from Scelimeninae.

**Figure 1.**
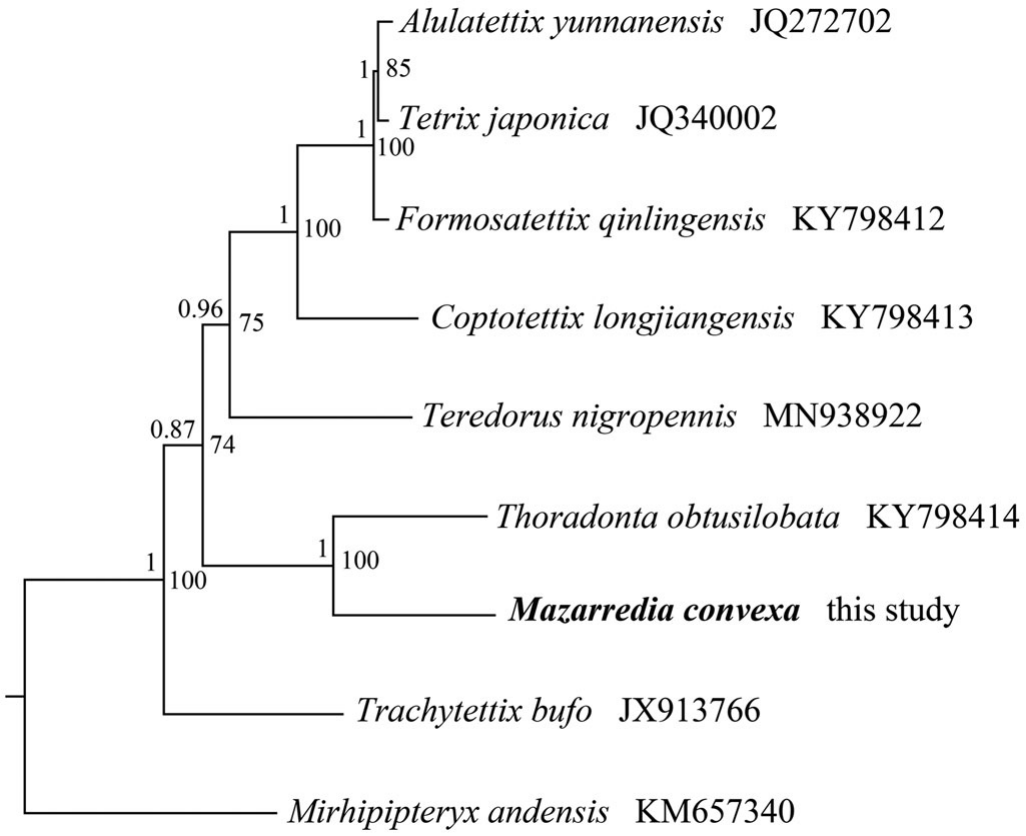
Phylogenetic tree obtained from ML and BI analysis based on 13 concatenated mitochondrial PCGs. Numbers on node are posterior probability (PP) and bootstrap value (BV).

## Data Availability

The data that support the findings of this study are openly available in National Center for Biotechnology Information at https://www.ncbi.nlm.nih.gov/nuccore, reference number [MN938924].
